# Supplementary material for: Cell-type specific profiling of human entorhinal cortex at the onset of Alzheimer’s disease neuropathology
Source: bioRxiv. 2025 Feb 3:2024.12.31.630881. Originally published 2025 Jan 1. Preprint. [Version 3] doi: 10.1101/2024.12.31.630881 (PMC11722323; doi:10.1101/2024.12.31.630881)
Supplement: Supplement 4 [file media-4.pdf]

SAMPLE ID	BRAAK	CERAD	SEX	AGE	ApoE4	Brain Bank	Sample type	Method
C1	0	0	M	54	Yes	Idibell	FF/FFPE	snRNAseq/FANS/IF
C2	0	0	F	51	No	Idibell	FF/FFPE	snRNAseq/FANS/IF
C3	0	0	F	60	No	Idibell	FF/FFPE	snRNAseq/FANS/IF
C4	0	0	M	59	No	Idibell	FF/FFPE	snRNAseq/FANS/IF
C5	0	B	M	71	Yes	Idibell	FF/FFPE	snRNAseq/FANS/
C6	0	0	M	64	-	NBB	FF	ISH/IF
C7	0	0	F	64	-	NBB	FF	ISH/IF
C8	0	0	M	68	-	NBB	FF	ISH/IF
C9	0	0	F	55	-	Harvard	FFPE	ISH
C10	0	0	M	36	-	Harvard	FFPE	ISH
ADN1	II	0	M	70	No	Idibell	FF/FFPE	snRNAseq/FANS/IF
ADN2	II	B	F	72	No	Idibell	FF/FFPE	snRNAseq/FANS/IF
ADN3	II	B	M	71	No	Idibell	FF/FFPE	snRNAseq/FANS/IF
ADN4	II	B	M	60	No	Idibell	FF/FFPE	snRNAseq/FANS/IF
ADN5	II	B	F	78	Yes	Idibell	FF/FFPE	snRNAseq/FANS
ADN6	II	B	F	79	-	NBB	FF	ISH/IF
ADN7	I	B	M	66	-	NBB	FF	ISH/IF
ADN8	II	A	F	67	-	NBB	FF	ISH/IF
