# Supplementary material for: Cell-type specific profiling of human entorhinal cortex at the onset of Alzheimer’s disease neuropathology
Source: bioRxiv. 2025 Feb 3:2024.12.31.630881. Originally published 2025 Jan 1. Preprint. [Version 3] doi: 10.1101/2024.12.31.630881 (PMC11722323; doi:10.1101/2024.12.31.630881)
Supplement: Supplement 5 — Extended Data Fig. 1. Sample cohort and pathology validation. a) Immunofluorescence images of amyloid beta pathology (6E10, pink) and NFT (thioflavin S, green) in tissue sections from the different samples used for snRNAseq and FANS analysis. b) Western blot of ApoE4 (green) and total ApoE (red) in these same samples. Actin is used as loading control. Extended Data Fig. 2. Gene expression differences between control and ADN in different glia cells. a) Volcano plot showing the differentially expressed genes between control and ADN in microglia and dysregulated pathways in these cells. b) Volcano plot showing the differentially expressed genes between control and ADN in astrocytes and dysregulated pathways in these cells. c) Volcano plot showing the differentially expressed genes between control and ADN in OPC and dysregulated pathways in these cells. d) Volcano plot showing the differentially expressed genes between control and ADN in oligodendrocytes and dysregulated pathways in these cells. e) Venn diagram of the overlap between differentially expressed genes in oligodendrocytes in ADN and plaque-induced genes (OLIGs) identified in the mouse. Extended Data Fig. 3. OPC and oligodendrocyte subpopulations and their abundance in control and ADN. a) UMAP of the different OPC subpopulations and dot plot of the expression profiles for signature genes of prototypical subtypes. b) Upper panel: relative contribution of the different samples to a certain OPC subpopulation; lower panel: abundance of a certain OPC subtype in each sample. c) UMAP of the different oligodendrocyte subpopulations and dot plot of the expression profiles of prototypical subtypes signature genes. d) Upper panel: relative contribution of the different samples to a certain oligodendrocyte subpopulation; lower panel: abundance of a certain oligodendrocyte subtype in each sample. Extended Data Fig. 4. No differences in the abundance of different excitatory neuron subpopulations in early ADN. a) UMAP of th [file media-5.pdf]

**a**

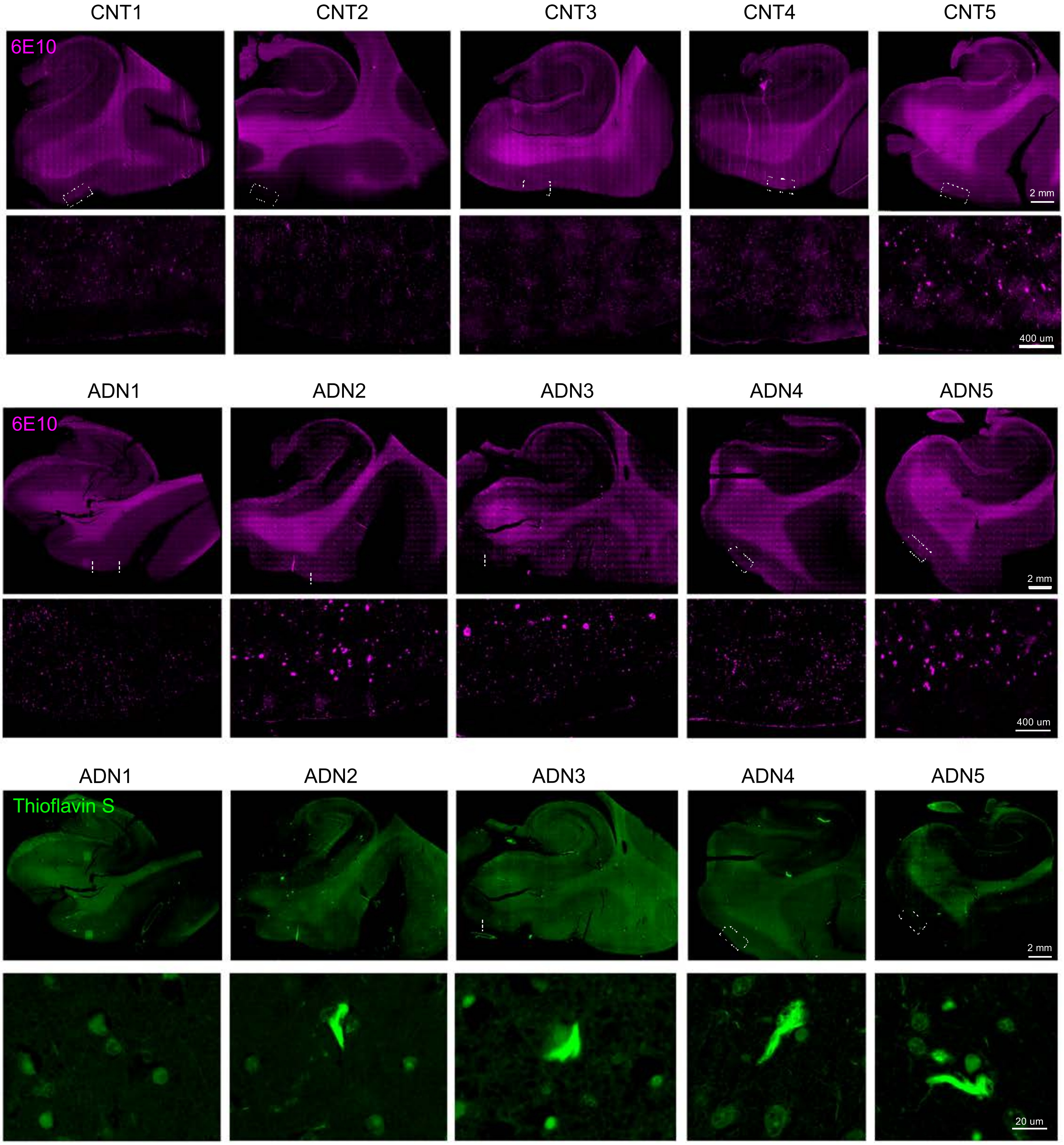
CNT1
CNT2
CNT3
CNT4
CNT5
6E10
2 mm
400 um
ADN1
ADN2
ADN3
ADN4
ADN5
6E10
2 mm
400 um
ADN1
ADN2
ADN3
ADN4
ADN5
Thioflavin S
2 mm
20 um

**b**

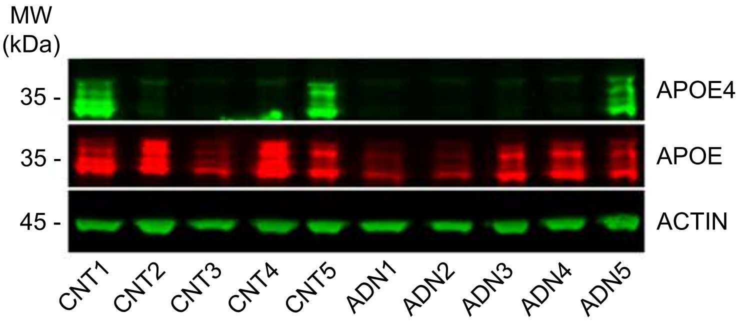
MW (kDa)
35 -
35 -
45 -
APOE4
APOE
ACTIN
CNT1
CNT2
CNT3
CNT4
CNT5
ADN1
ADN2
ADN3
ADN4
ADN5

Extended data Figure 2

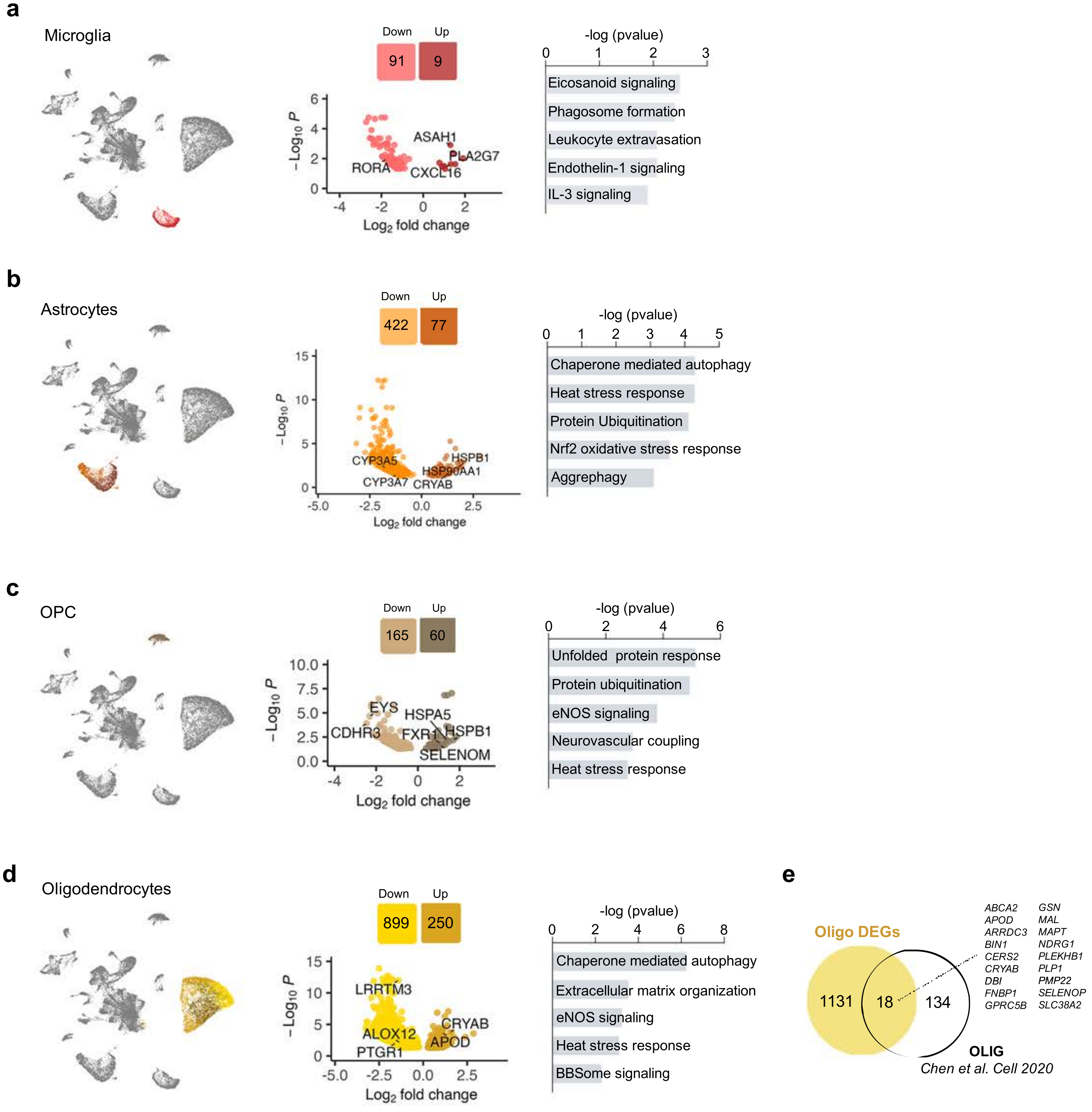

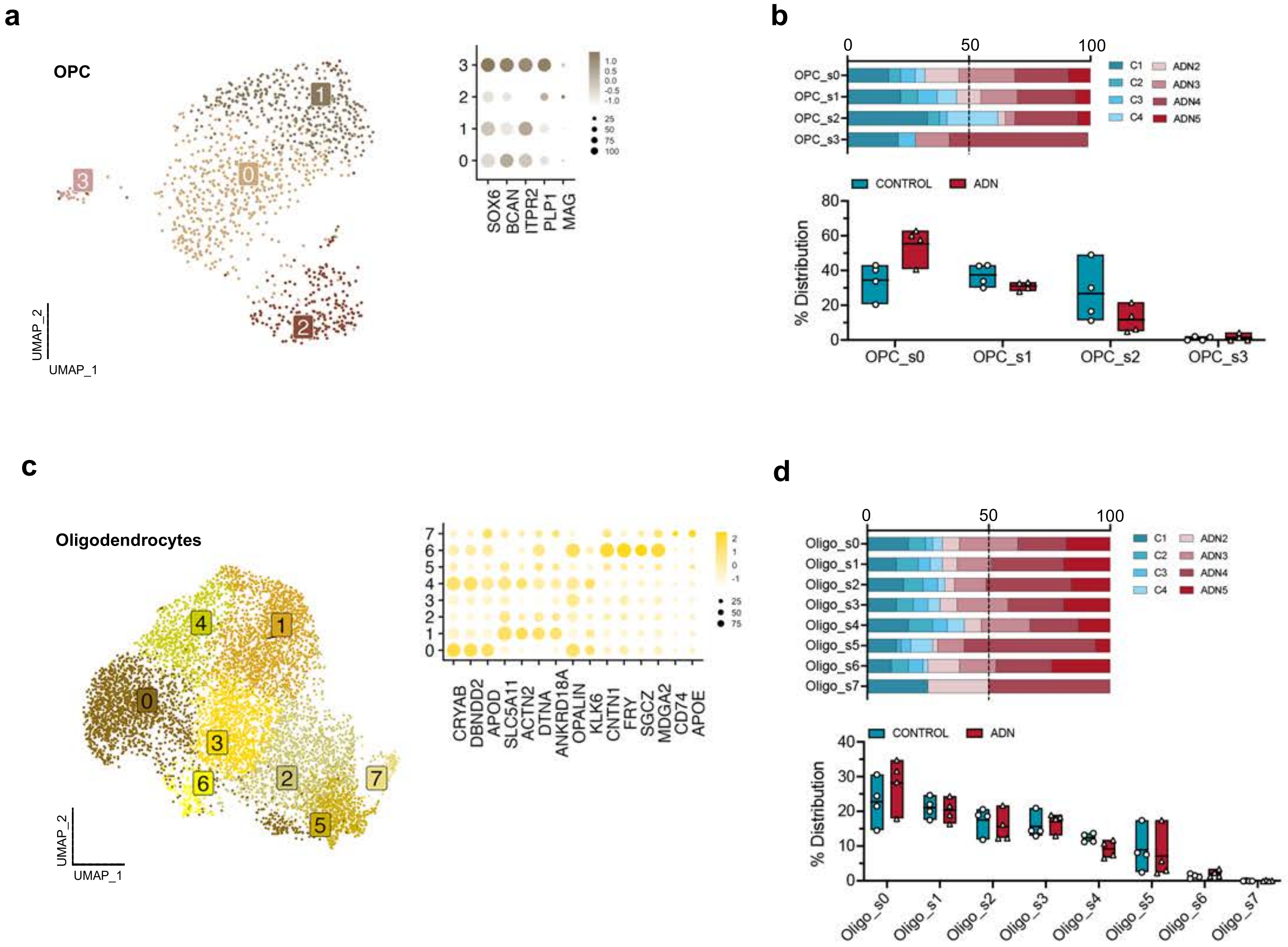
a
OPC
0
1
2
3
UMAP_2
UMAP_1
SOX6
BCAN
ITPR2
PLP1
MAG
b
OPC_s0
OPC_s1
OPC_s2
OPC_s3
C1
C2
C3
C4
ADN2
ADN3
ADN4
ADN5
CONTROL
ADN
% Distribution
c
Oligodendrocytes
CRYAB
DBNDD2
APOD
SLC5A11
ACTN2
DTNA
ANKRD18A
OPALIN
KLK6
CNTN1
FRY
SGCZ
MDGA2
CD74
APOE
d
Oligo_s0
Oligo_s1
Oligo_s2
Oligo_s3
Oligo_s4
Oligo_s5
Oligo_s6
Oligo_s7

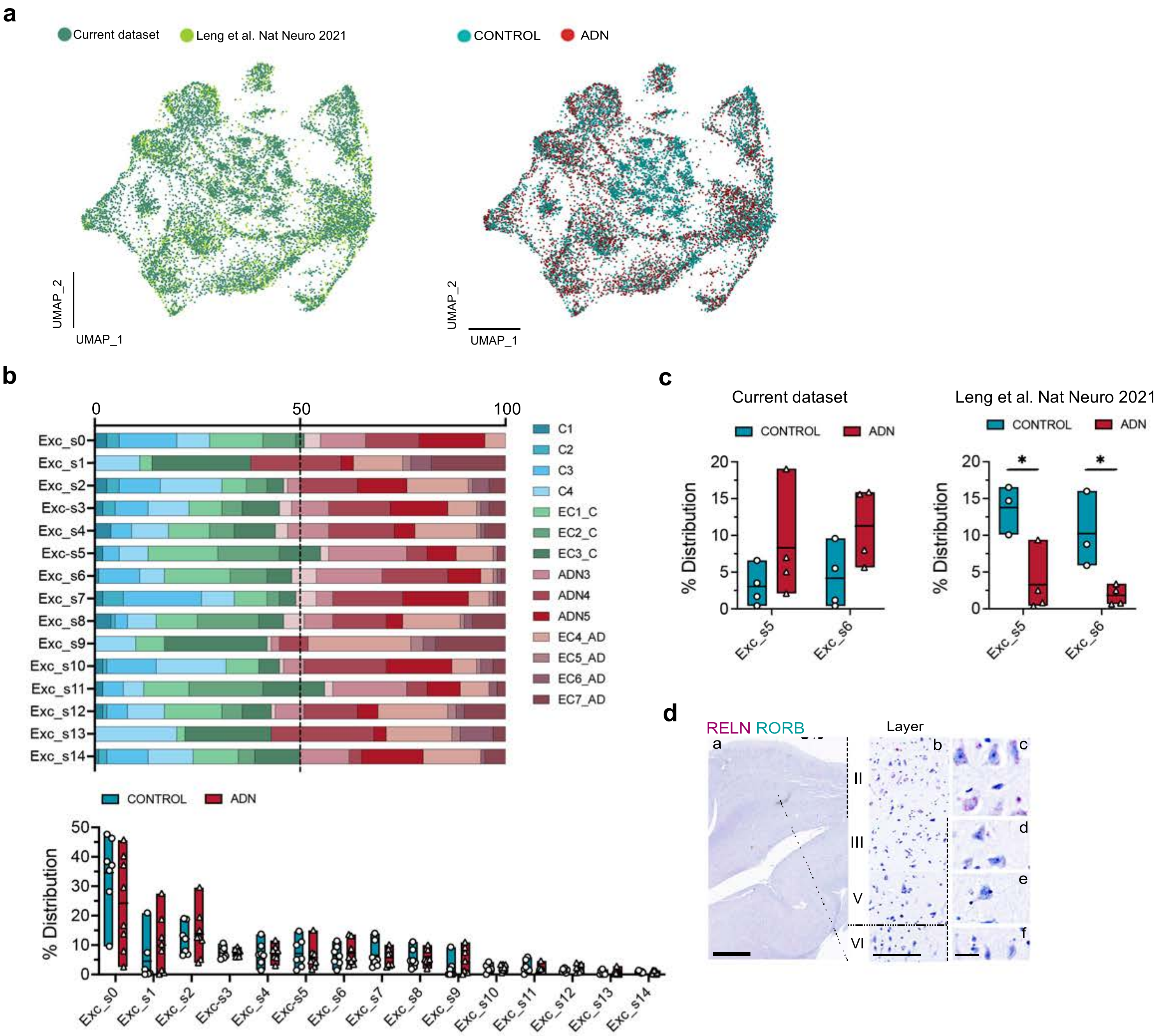
a
Current dataset
Leng et al. Nat Neuro 2021
CONTROL
ADN
UMAP_2
UMAP_1
b
Exc_s0
Exc_s1
Exc_s2
Exc-s3
Exc_s4
Exc-s5
Exc_s6
Exc_s7
Exc_s8
Exc_s9
Exc_s10
Exc_s11
Exc_s12
Exc_s13
Exc_s14
C1
C2
C3
C4
EC1_C
EC2_C
EC3_C
ADN3
ADN4
ADN5
EC4_AD
EC5_AD
EC6_AD
EC7_AD
% Distribution
c
Current dataset
Leng et al. Nat Neuro 2021
d
RELN RORB
Layer
II
III
V
VI

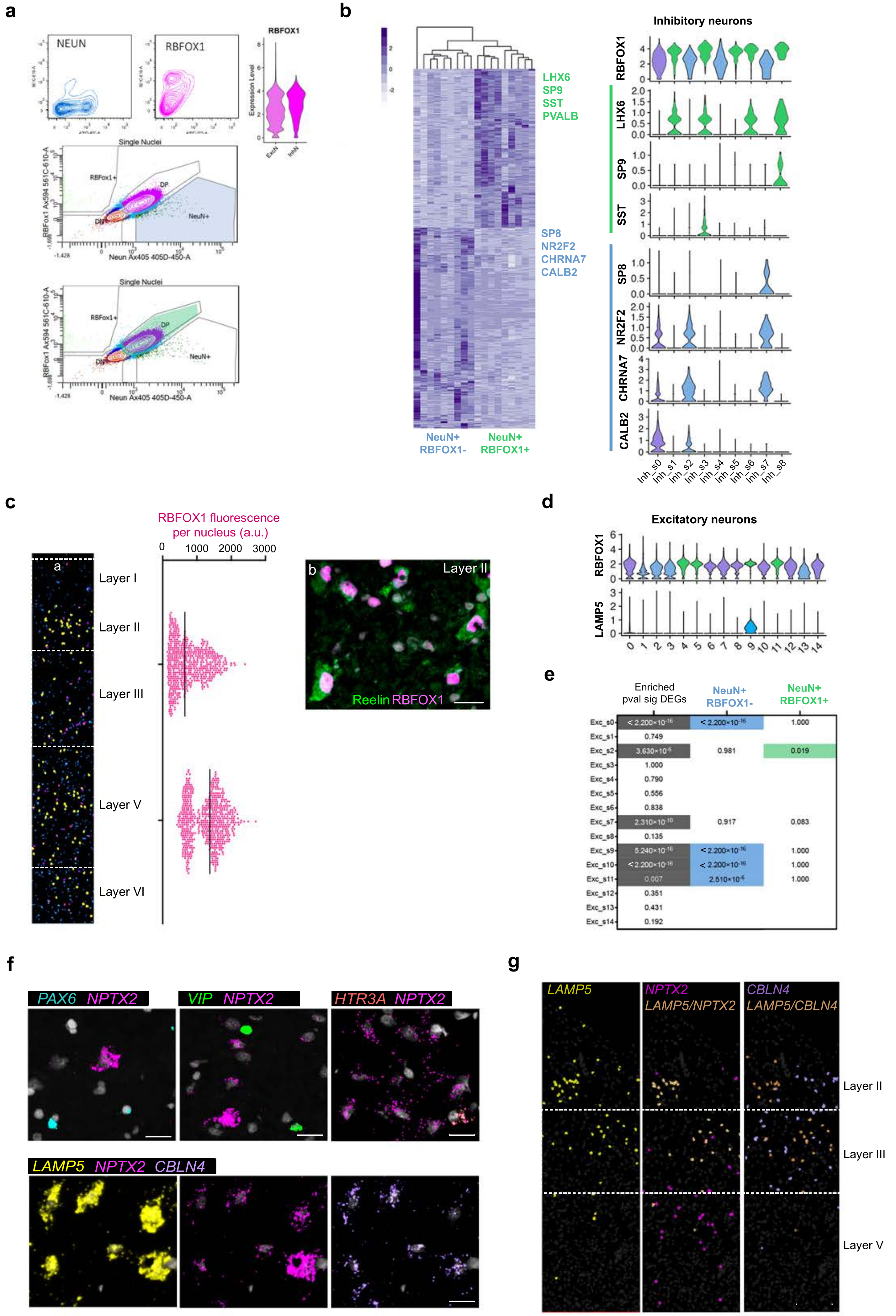


| | Enriched pval sig DEGs | NeuN+ RBFOX1- | NeuN+ RBFOX1+ |
|---|---|---|---|
| Exc_s0 | $< 2.200\times10^{-16}$ | $< 2.200\times10^{-16}$ | 1.000 |
| Exc_s1 | 0.749 | | |
| Exc_s2 | $3.630\times10^{-6}$ | 0.981 | 0.019 |
| Exc_s3 | 1.000 | | |
| Exc_s4 | 0.790 | | |
| Exc_s5 | 0.556 | | |
| Exc_s6 | 0.838 | | |
| Exc_s7 | $2.310\times10^{-10}$ | 0.917 | 0.083 |
| Exc_s8 | 0.135 | | |
| Exc_s9 | $5.240\times10^{-16}$ | $< 2.200\times10^{-16}$ | 1.000 |
| Exc_s10 | $< 2.200\times10^{-16}$ | $< 2.200\times10^{-16}$ | 1.000 |
| Exc_s11 | 0.007 | $2.510\times10^{-6}$ | 1.000 |
| Exc_s12 | 0.351 | | |
| Exc_s13 | 0.431 | | |
| Exc_s14 | 0.192 | | |

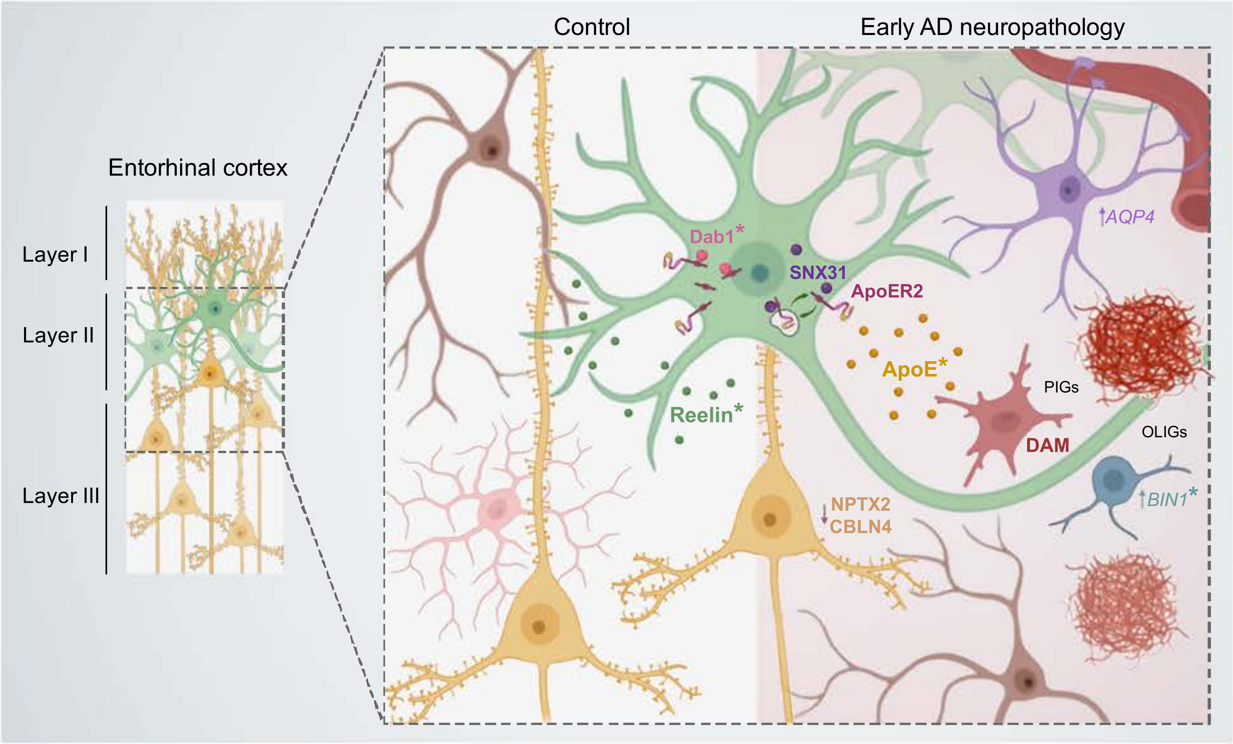
Control
Early AD neuropathology
Entorhinal cortex
Layer I
Layer II
Layer III
Dab1*
SNX31
ApoER2
ApoE*
Reelin*
PIGs
DAM
OLIGs
AQP4
BIN1*
NPTX2
CBLN4
