# Supplementary material for: Cell-type specific profiling of human entorhinal cortex at the onset of Alzheimer’s disease neuropathology
Source: bioRxiv. 2025 Jan 1:2024.12.31.630881. Preprint. [Version 1] doi: 10.1101/2024.12.31.630881 (PMC11722323; doi:10.1101/2024.12.31.630881)
Supplement: Supplementary file 1 [file media-1.pdf]

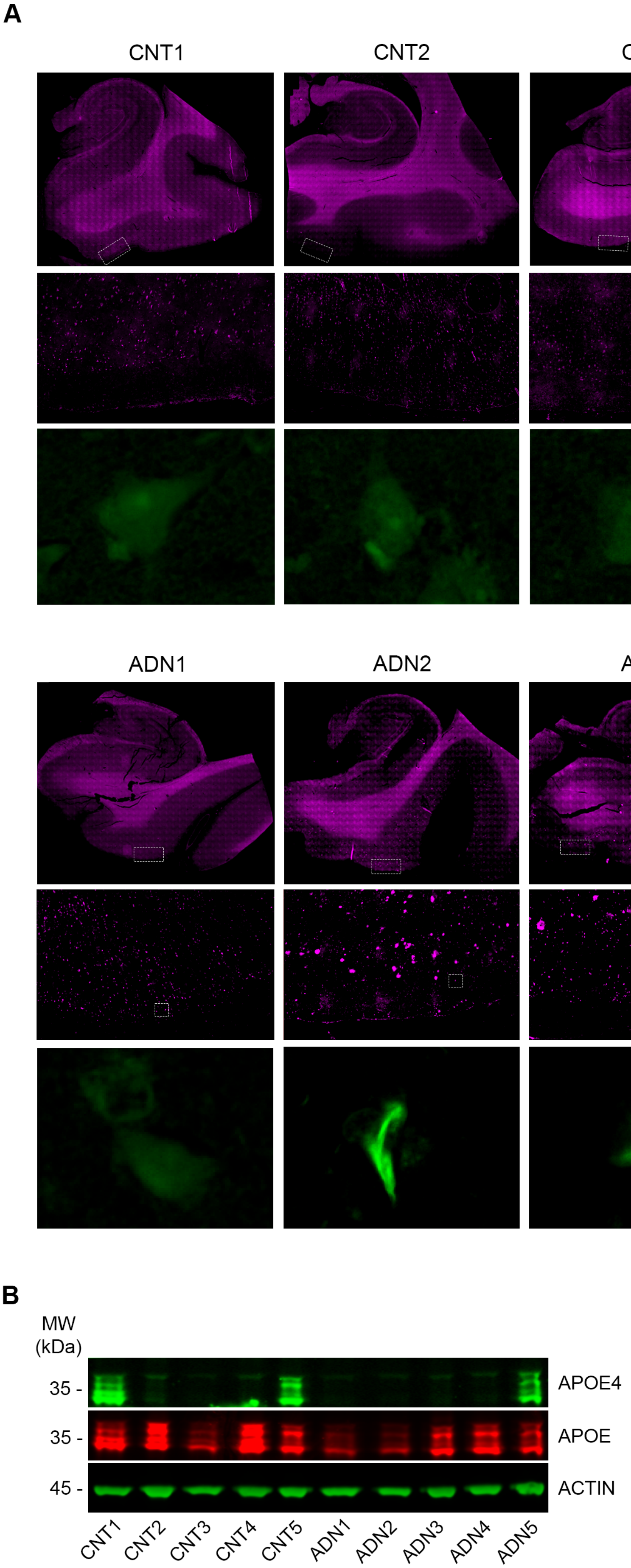

Supplementary Figure 1. Sample cohort and pathology validation. A) Immunofluorescence images of amyloid beta pathology (pink) and NFT (thioflavin S, green) in tissue sections from the different samples used for snRNAseq and FANS analysis. **B)** Western blot of ApoE4 and total ApoE in these same samples. Actin is used as loading control.

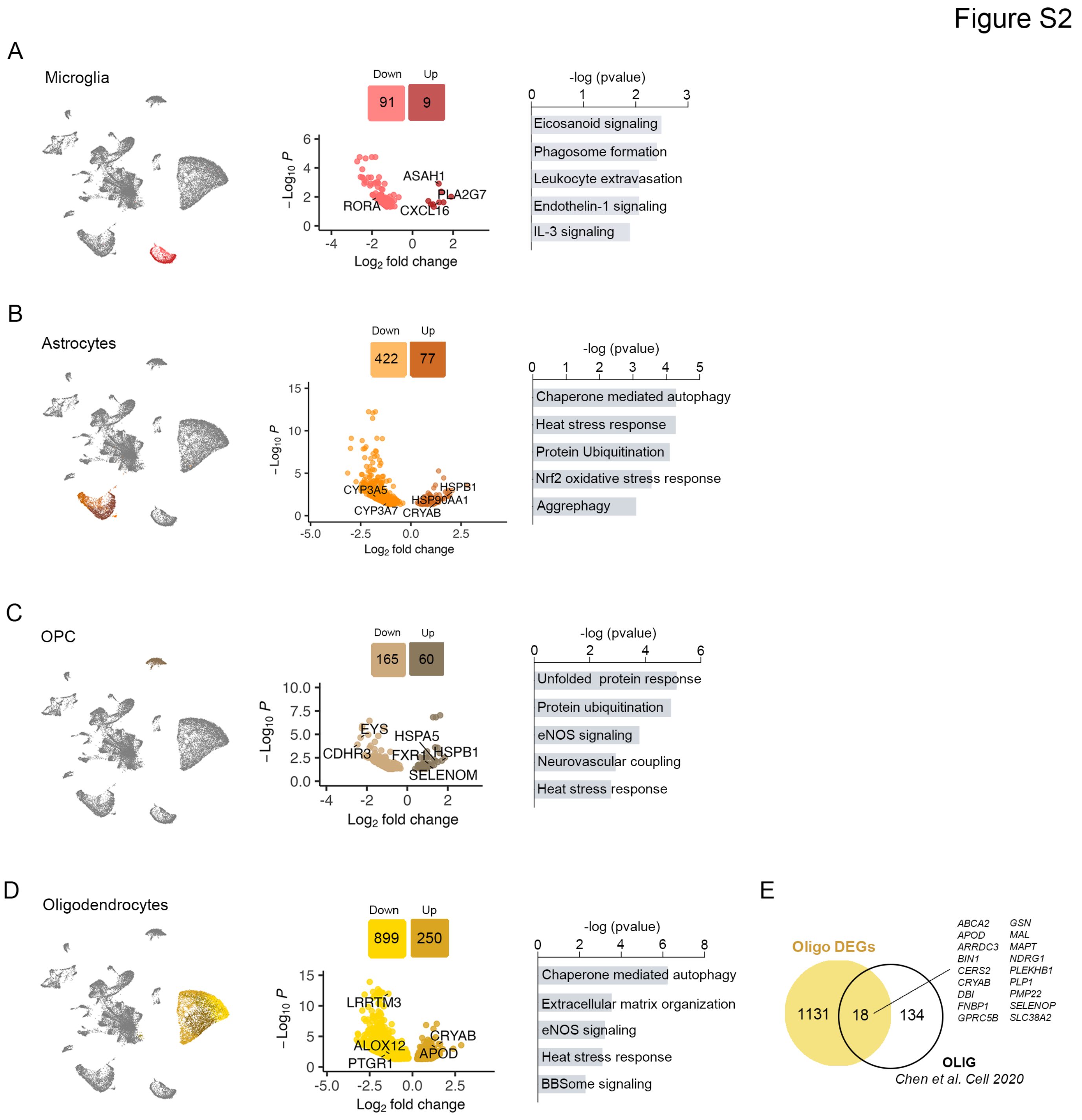

Supplementary Figure 2. Gene expression differences between control and ADN in different glia cells. A) Volcano plot showing the differentially expressed genes between control and ADN in microglia and dysregulated pathways in these cells. **B)** Volcano plot showing the differentially expressed genes between control and ADN in astrocytes and dysregulated pathways in these cells. **C)** Volcano plot showing the differentially expressed genes between control and ADN in OPC and dysregulated pathways in these cells. **D)** Volcano plot showing the differentially expressed genes between control and ADN in oligodendrocytes and dysregulated pathways in these cells. **E)** Venn diagram of the overlap between differentially expressed genes in oligodendrocytes in ADN and plaque-induced genes (OLIGs) identified in the mouse.

Figure S3

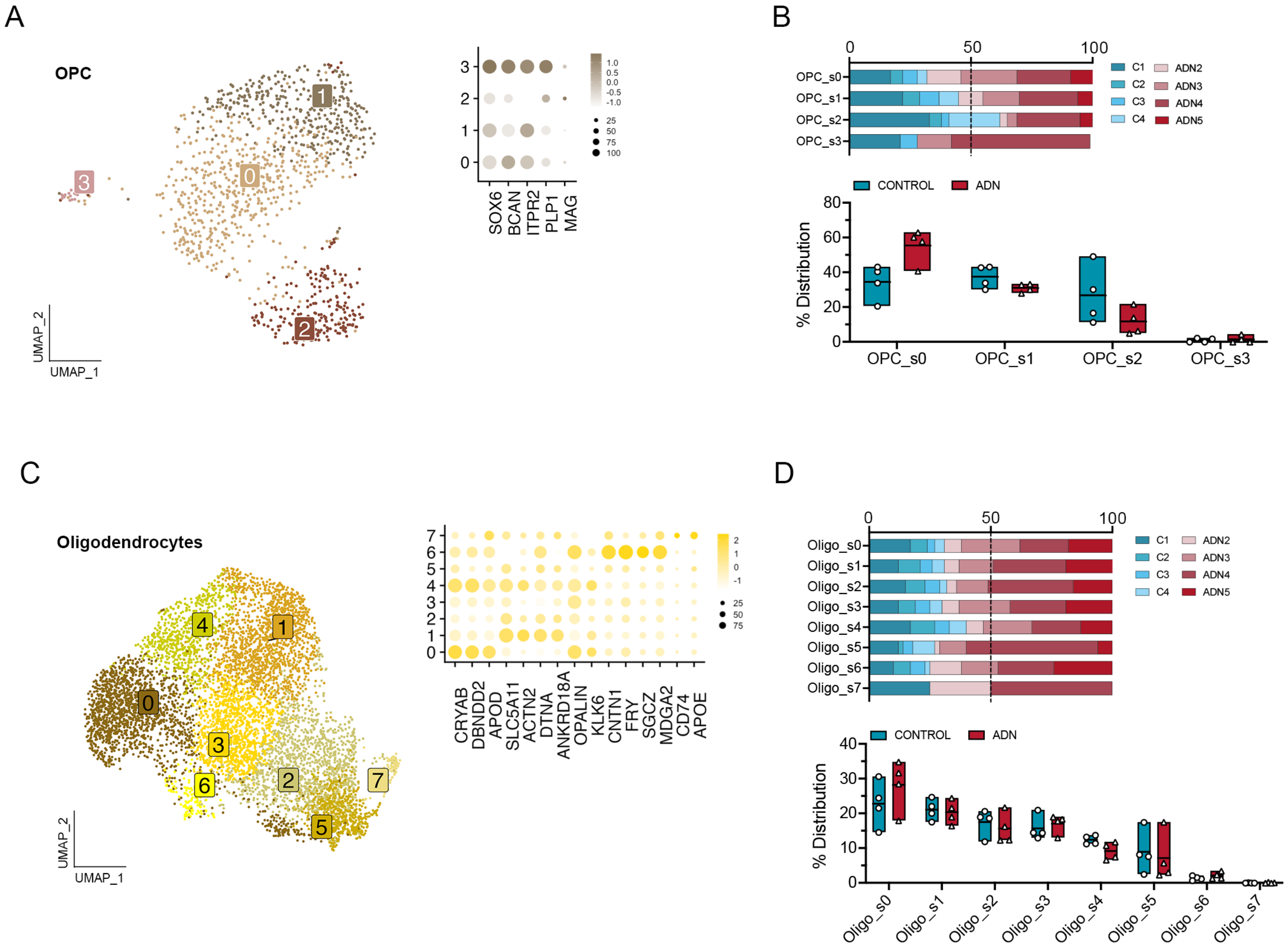

Supplementary Figure 3. OPC and oligodendrocyte subpopulations and their abundance in control and ADN. A) UMAP of the different OPC subpopulations and dot plot of the expression profiles of prototypical subtypes signature genes. **B)** Upper panel: relative contribution of the different samples to a certain OPC subpopulation; lower panel: abundance of a certain OPC subtype in each sample. **C)** UMAP of the different oligodendrocyte subpopulations and dot plot of the expression profiles of prototypical subtypes signature genes. **D)** Upper panel: relative contribution of the different samples to a certain oligodendrocyte subpopulation; lower panel: abundance of a certain oligodendrocyte subtype in each sample.

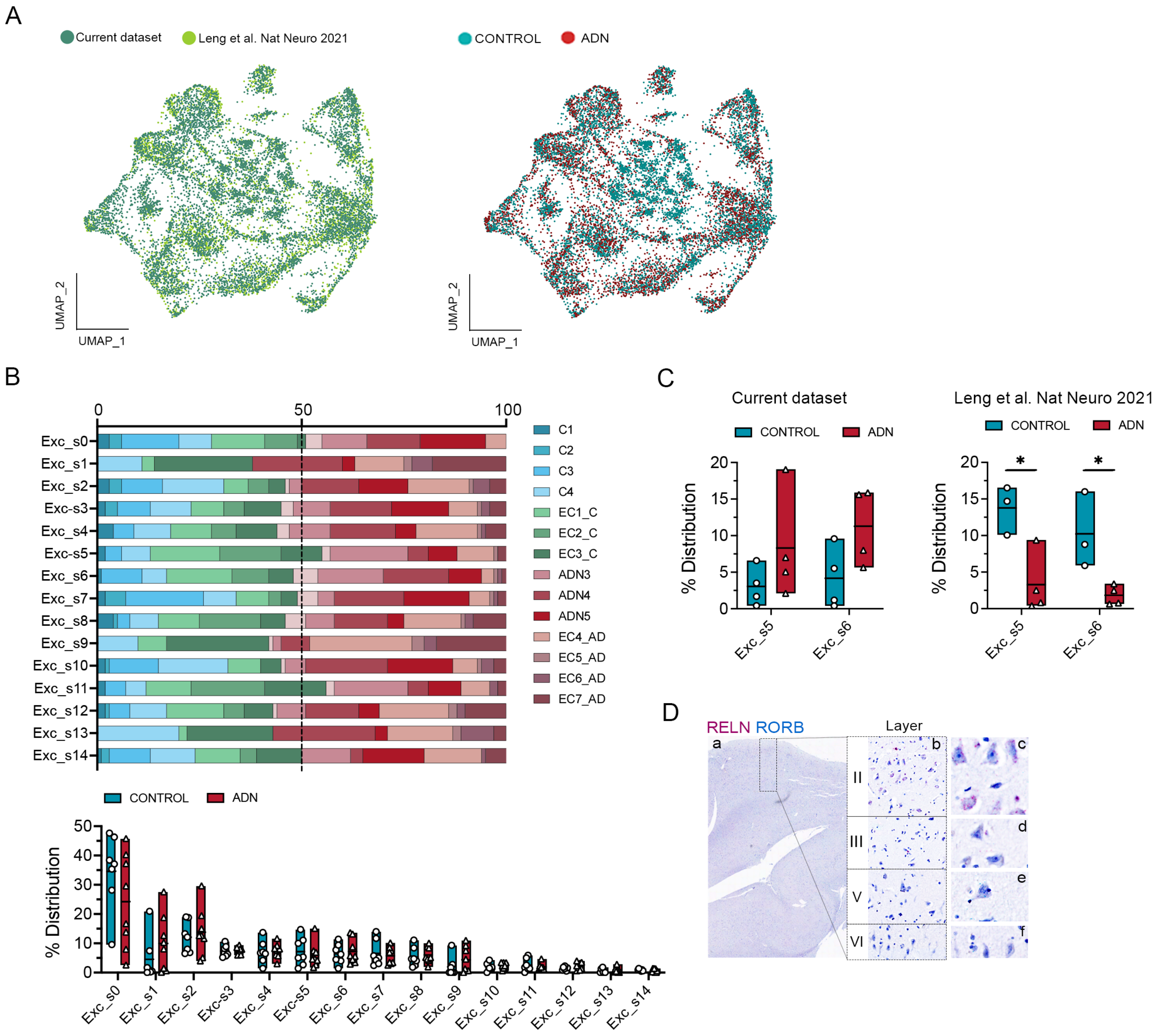

Supplementary Figure 4. No differences in the abundance of different excitatory neuron subpopulations in early ADN. **A)** UMAP of the integration of our dataset (blue) with Leng et al. (green) and the integration between control (blue) and ADN samples (red). **B)** Right panel: relative contribution of the different samples to a certain excitatory neuron subpopulation; right panel: abundance of a certain excitatory neuron subtype in each sample. **C)** Relative abundance of the RORB+ excitatory neuron subpopulation in our dataset and in Leng et al. **D)** In-situ hybridization images of RELN (red) and RORB (blue) in the human EC.

Figure S5

A

B

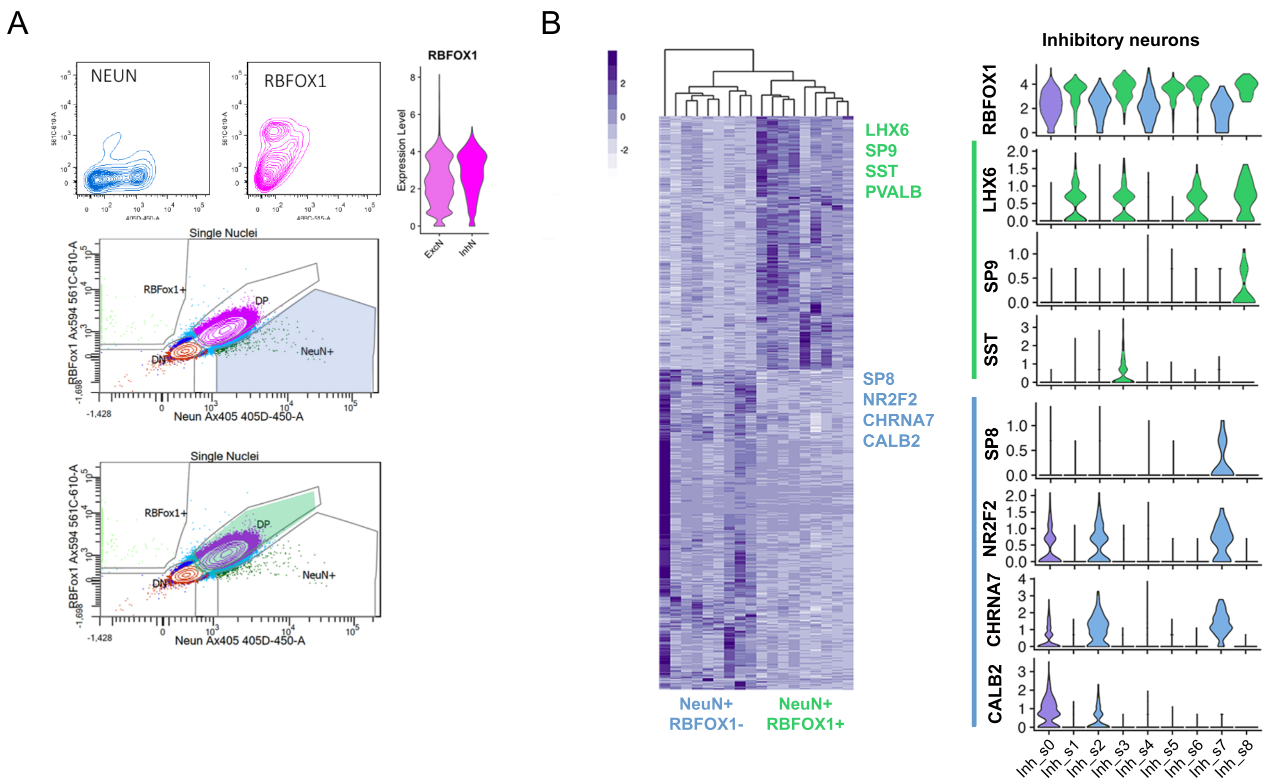

C

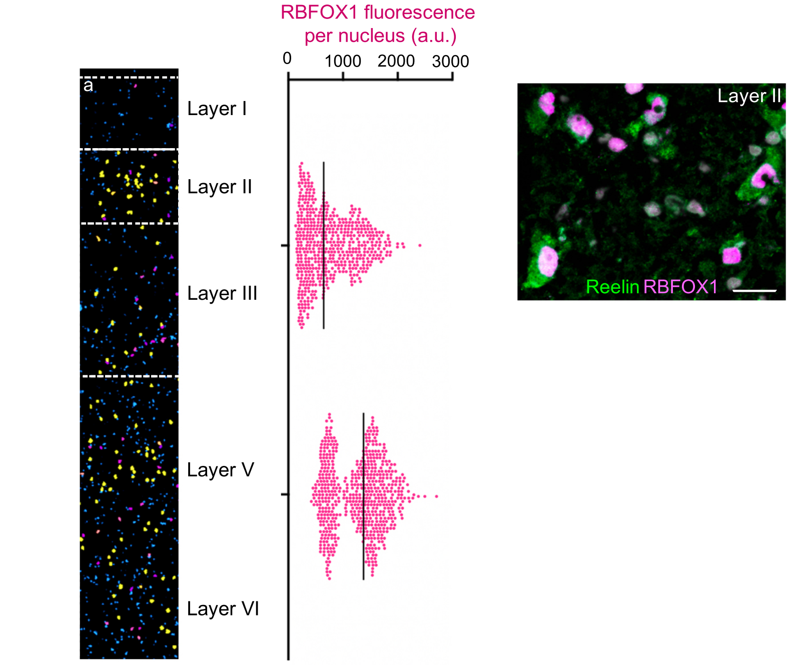

D

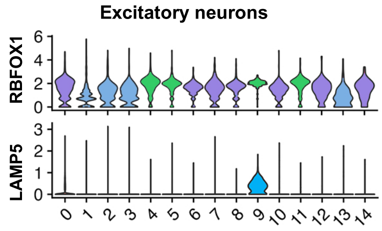

E

	Enriched pval sig DEGs	NeuN+ RBFOX1-	NeuN+ RBFOX1+
Exc_s0	$< 2.200\times10^{-16}$	$< 2.200\times10^{-16}$	1.000
Exc_s1	0.749		
Exc_s2	3.630×10^{-6}	0.981	0.019
Exc_s3	1.000		
Exc_s4	0.790		
Exc_s5	0.556		
Exc_s6	0.838		
Exc_s7	2.310×10^{-10}	0.917	0.083
Exc_s8	0.135		
Exc_s9	5.240×10^{-16}	$< 2.200\times10^{-16}$	1.000
Exc_s10	$< 2.200\times10^{-16}$	$< 2.200\times10^{-16}$	1.000
Exc_s11	0.007	2.510×10^{-6}	1.000
Exc_s12	0.351		
Exc_s13	0.431		
Exc_s14	0.192		

F

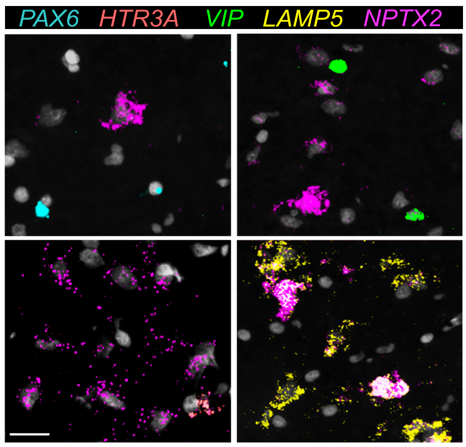

G

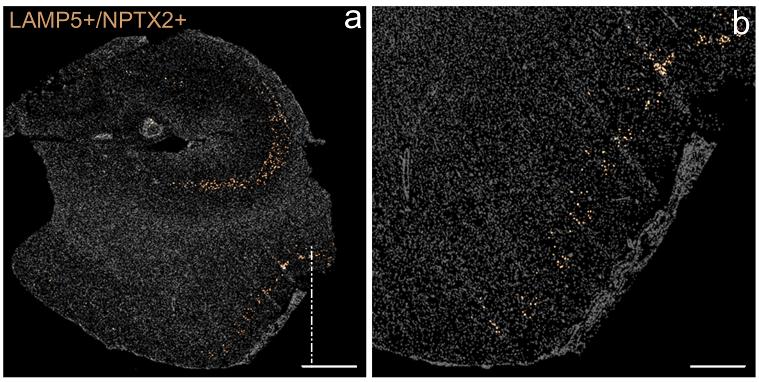

Supplementary Figure 5. Gating strategy and neuron subpopulations sorted by FANS. A) Gating strategy used for FANS of the different neuronal populations. **B)** Heatmap of the differentially expressed genes between NeuN+/RBFOX1- and NeuN+/RBFOX1+ subpopulations and violin plots of their expression in the different inhibitory neuron subpopulations from snRNAseq. **C)** a. RBFOX1 signal intensity map of the different EC layers and RBFOX1fluorescence intensity quantifications for each EC layer. b. RBFOX1 levels in Reelin positive neurons in the ECII. Scale bar 20 µm. **D)** Violin plots of RBFOX1 and LAMP5 expression in the different excitatory neuron subpopulations from snRNAseq. **E)** Heatmap of the Wilcoxon test results for the enrichment of genes from the different excitatory neuron subpopulations in pvalue significant DEGs between the NeuN+/RBFOX1- and NeuN+/RBFOX1+ (grey values) and for the enrichment of genes from the different excitatory neuron subpopulation based on Log2FC DEGs between NeuN+/RBFOX1- and NeuN+/RBFOX1+ (blue and green values). **F)** ISH images of PAX6, VIP, LAMP5 and NPTX2 co-localization in the human EC. Scale bar 20 µm. **G)** Spatial localization of LAMP5+/NPTX2+ cells in the human EC. Scale bar 2 mm (a) and 800 µm (b).

SAMPLE ID	BRAAK	CERAD	SEX	AGE	ApoE4	Brain Bank	Sample type	Method
C1	0	0	M	54	Yes	Idibell	FF/FFPE	snRNAseq/FANS/IF
C2	0	0	F	51	No	Idibell	FF/FFPE	snRNAseq/FANS/IF
C3	0	0	F	60	No	Idibell	FF/FFPE	snRNAseq/FANS/IF
C4	0	0	M	59	No	Idibell	FF/FFPE	snRNAseq/FANS/IF
C5	0	B	M	71	Yes	Idibell	FF/FFPE	snRNAseq/FANS/
C6	0	0	M	64	-	NBB	FF	ISH/IF
C7	0	0	F	64	-	NBB	FF	ISH/IF
C8	0	0	M	68	-	NBB	FF	ISH/IF
C9	0	0	F	55	-	Harvard	FFPE	ISH
C10	0	0	M	36	-	Harvard	FFPE	ISH
ADN1	II	0	M	70	No	Idibell	FF/FFPE	snRNAseq/FANS/IF
ADN2	II	B	F	72	No	Idibell	FF/FFPE	snRNAseq/FANS/IF
ADN3	II	B	M	71	No	Idibell	FF/FFPE	snRNAseq/FANS/IF
ADN4	II	B	M	60	No	Idibell	FF/FFPE	snRNAseq/FANS/IF
ADN5	II	B	F	78	Yes	Idibell	FF/FFPE	snRNAseq/FANS
ADN6	II	B	F	79	-	NBB	FF	ISH/IF
ADN7	I	B	M	66	-	NBB	FF	ISH/IF
ADN8	II	A	F	67	-	NBB	FF	ISH/IF

Supplementary Table 1. Sample cohort information.
